# Risk Factors and Protective Factors against Ventilator-Associated Pneumonia—A Single-Center Mixed Prospective and Retrospective Cohort Study

**DOI:** 10.3390/jpm12040597

**Published:** 2022-04-08

**Authors:** Jarosław Pawlik, Lucyna Tomaszek, Henryk Mazurek, Wioletta Mędrzycka-Dąbrowska

**Affiliations:** 1 Faculty of Medicine and Health Sciences, Andrzej Frycz Modrzewski Krakow University, 30-705 Krakow, Poland; jarek.pawlik@yahoo.com (J.P.); or ltomaszek@igrabka.edu.pl (L.T.); 2 National Institute of Tuberculosis and Lung Diseases, 34-700 Rabka-Zdroj, Poland; 3 Department of Pneumonology and Cystic Fibrosis, National Institute of Tuberculosis and Lung Diseases, 34-700 Rabka-Zdroj, Poland; hmazurek@igrabka.edu.pl; 4 Institute of Health, State University of Applied Sciences in Nowy Sącz, 33-300 Nowy Sącz, Poland; 5 Department of Anesthesiology Nursing & Intensive Care, Faculty of Health Sciences, Medical University of Gdansk, 80-211 Gdansk, Poland

**Keywords:** ventilator-associated pneumonia, bundle, risk factors, subglottic secretion suction, continuous control pressure

## Abstract

Introduction: Understanding the factors associated with the development of ventilator-associated pneumonia (VAP) in critically ill patients in the intensive care unit (ICU) will allow for better prevention and control of VAP. The aim of the study was to evaluate the incidence of VAP, as well as to determine risk factors and protective factors against VAP. Design: Mixed prospective and retrospective cohort study. Methods: The cohort involved 371 critically ill patients who received standard interventions to prevent VAP. Additionally, patients in the prospective cohort were provided with continuous automatic pressure control in tapered cuffs of endotracheal or tracheostomy tubes and continuous automatic subglottic secretion suction. Logistic regression was used to assess factors affecting VAP. Results: 52 (14%) patients developed VAP, and the incidence density of VAP per 1000 ventilator days was 9.7. The median days to onset of VAP was 7 [4; 13]. Early and late onset VAP was 6.2% and 7.8%, respectively. According to multivariable logistic regression analysis, tracheotomy (OR = 1.6; CI 95%: 1.1 to 2.31), multidrug-resistant bacteria isolated in the culture of lower respiratory secretions (OR = 2.73; Cl 95%: 1.83 to 4.07) and ICU length of stay >5 days (OR = 3.32; Cl 95%: 1.53 to 7.19) were positively correlated with VAP, while continuous control of cuff pressure and subglottic secretion suction used together were negatively correlated with VAP (OR = 0.61; Cl 95%: 0.43 to 0.87). Conclusions: Tracheotomy, multidrug-resistant bacteria, and ICU length of stay >5 days were independent risk factors of VAP, whereas continuous control of cuff pressure and subglottic secretion suction used together were protective factors against VAP.

## 1. Introduction

Ventilator-associated pneumonia (VAP) in the intensive care unit (ICU) is a complex and multifactorial clinical condition connected with high morbidity (4–61%) and mortality (40–43%) as well as considerable treatment costs [1,2,3,4]. The length of stay of a patient with VAP in the ward usually fluctuates between 10 and 21 days. It has been documented that a patient with VAP stays in the hospital from 6 to 13 days longer than a patient without such an infection [5], and this increases the economic burden on the unit [6]. It has been estimated that, for the ICU, the cost of treating a patient with VAP is almost three times higher than that of a patient without the condition [4].

Much research has studied the clinical epidemiology of VAP in ICUs. Analyzing the results of prospective observational studies, which involved patients from 27 ICUs in 9 European countries, Koulenti et al. [7] identified the dominant microorganisms responsible for nosocomial pneumonia, including VAP. In countries, such as Spain, France, Belgium and Ireland, Staphylococcus aureus was the dominant isolate from the secretion of the lower respiratory tract, while in Italy and Portugal, it was Pseudomonas aeruginosa, and in Germany, Escherichia coli. Acinetobacter baumannii was most prevalent in Greece and Turkey [7], as was in Chinese ICUs [8]. In Poland, in the study conducted by Wałaszek et al. [9], *Enterobacteriaceae* and non-fermenting Gram-negative *bacilli* were the main etiological factors responsible for VAP. Many of the VAP-causing isolates demonstrated multidrug resistance [5,10] and were associated with a higher initial antibiotic treatment failure and an increased mortality risk [10].

It is very important to explain the risk factors of VAP in order to develop more effective prevention and control. Based on a review of literature, in which the results of retrospective and prospective clinical trials from recent years were published, Wu et al. [11] identified 13 potential risk factors for VAP: advanced age and male, increased mechanical ventilation time, prolonged length of hospital stay, disorders of consciousness, burns, comorbidities, prior antibiotic therapy, invasive operations, gene polymorphisms, smoking, intra-abdominal hypertension and hyperoxemia. The cited authors noted that the above-mentioned independent risk factors may also influence each other. In their study, Xu et al. [12] also indicated the following risk factors: the number of central venous catheters, the duration of maintaining the catheter in the urinary bladder, the number of antibiotics administered, and the use of corticosteroids before mechanical ventilation. In the research conducted by Lee et al. [13], VAP was strongly correlated with the severity of an injury, the use of vasopressors and the insertion of a nasogastric tube.

Reducing the undesirable effects of mechanical ventilation is possible thanks to the implementation of VAP prevention bundle in clinical practice. The core bundle involves the proper positioning of the patient in bed (head-of-bed elevation to 30–45 degrees), the appropriate sedation (daily sedation vacation and the assessment of readiness to extubate) and oral care (oral hygiene with chlorhexidine). The prevention of peptic ulcer disease and deep vein thrombosis, although not directly related to VAP prevention, is also part of the core bundle [14]. In the VAP prevention guidelines for Spanish ICUs (“Pneumonia Zero”), Álvarez-Lerma et al. [15] also recommend measures to reduce the risk of aspiration of contaminated secretions from the upper respiratory tract into the bronchial tree, through microchannels around the cuff of an endotracheal or tracheostomy tube. Maintaining adequate pressure in the tubing cuff and suctioning secretions from above the cuff can help reduce this risk. Researchers pay attention to the effectiveness of both single interventions and comprehensive measures in the prevention of VAP [16,17,18,19,20]. However, Colombo et al. [20] note, that due to, among others, the lack of a gold standard in VAP diagnostics, the evidence for the effectiveness of pharmacological and non-pharmacological interventions in reducing the incidence of VAP is unreliable.

Given the above considerations, it seems important to clearly identify high-risk subgroups of mechanically ventilated patients for the safe and effective implementation of preventive measures against VAP. Therefore, the aim of the study was to evaluate the incidence of VAP in critically ill patients in the intensive care unit, as well as to determine risk factors and protective factors against VAP.

## 2. Material and Methods

### 2.1. Study Design and Setting

**Figure 1:**
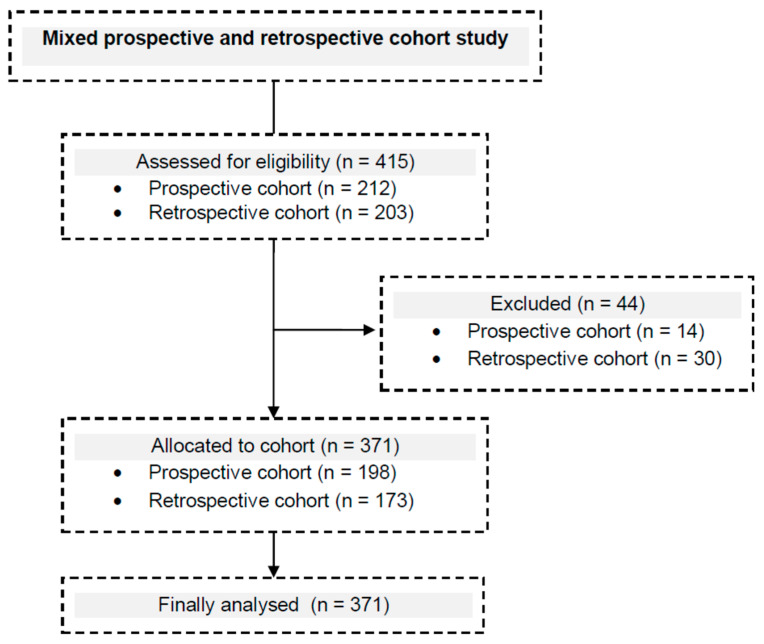
Flow diagram.

This single-center study involved 371 critically ill patients (Figure 1) treated in the 8-bed Clinical Department of Anesthesiology and Intensive Therapy of the St. Raphael Hospital in Kraków at the University Department of Anesthesiology, Intensive Therapy and Emergency Medicine of the Faculty of Medicine and Health Sciences of the Andrzej Frycz Modrzewski Kraków University. In order to improve inference, data were collected prospectively (from 1 June 2018 to 1 July 2019) and retrospectively (from 1 May 2017 to 30 April 2018). The follow-up protocols were similar in both cohorts. The study protocol was approved by the Bioethical Committee of the Andrzej Frycz Modrzewski Kraków University (opinion no. KBKA/34/O/2018) in accordance with the Declaration of Helsinki. The informed consent requirement in the prospective cohort was waived because the VAP prevention bundle was a life-saving therapy. The study was registered with Clinicaltrails.gov under the number NCT04038814, https://clinicaltrials.gov/ct2/show/NCT04038814 (accessed on 9 September 2021). The study was reported in accordance with STROBE guidelines [21].

### 2.2. Participants

Intubated patients older than 18 years whose duration of mechanical ventilation was over 48 h qualified for both cohorts. Percutaneous tracheostomy (Blue Rhino Ciaglia 2) [22] was performed in all patients requiring prolonged mechanical ventilator support (for more than 14 days). In addition, in the prospective cohort, patients admitted to ICUs already intubated with a standard tube for less than 12 h, required re-intubation. In those patients, a tube with a tapered cuff and channel for subglottic secretion suction was used.

Pregnant patients were excluded from the study. Other exclusion criteria were nasally intubated patients or those intubated for more than 12 h before ICU admission, as well as those undergoing a tracheostomy upon ICU admission.

### 2.3. VAP Prevention Bundle

In both cohorts, each mechanically ventilated patient received interventions to prevent VAP. Simple interventions, as a part of ventilator bundle care, are shown in Table 1.

### 2.4. Variables

For each subject, the following variables were recorded: age, gender, body mass index, Acute Physiology and Chronic Health Evaluation (APACHE) II score [23] and estimated risk of death (%), total VAP incidence, early-onset VAP incidence (developed during the first 4 days of mechanical ventilation), late-onset VAP incidence (developed after 4 days of mechanical ventilation), time to VAP, length of ventilator use, ICU length of stay, ICU mortality, the results of a lower respiratory tract specimen culture. Type of admission, comorbidities and type of artificial airway were also collected.

VAP was recognized according to the Chest Echocardiography and Procalcitonin Pulmonary Infection Score (CEPPIS) by the anesthesiologist on duty. VAP was suspected if patients were at least 48 h following intubation and initiation of mechanical ventilatory support and CEPPIS was >5 points. CEPPIS is based on the following variables:

- Body temperature (≥38.5 and <38.9 °C = 1 point; ≥39 and <36 °C = 2 points),
- Procalcitonin (≥0.5 and <1 ng/mL = 1 point; ≥1 ng/mL = 2 points),
- Purulent tracheal secretions = 2 points,
- Positive endotracheal aspirate (>10^4^ colony–forming units/mL) = 2 points,
- Positive infiltrates on chest echograph (sub-pleural echo-poor region or more with tissue-like echo texture) = 2 points,
- Oxygenation PaO_2_/FiO_2_ ≤ 240 and absence of acute respiratory distress syndrome = 2 points.

Microbial investigation in VAP was mainly based on the culture of samples obtained from lower respiratory tract noninvasively via tracheal aspiration using a closed suction system. In patients suffered from severe atelectasis, samples for sputum culture were obtained invasively with bronchoalveolar lavage. Furthermore, for all patients, hospital radiologists assessed and described chest X-rays or computed tomography, which were performed on admission to the ICU and when the ultrasound image suggested pneumonia.

### 2.5. Outcomes

The primary end-point variable was the incidence of VAP. The secondary outcomes were factors associated with VAP.

### 2.6. Statistics

Categorical variables were expressed as total numbers and percentages, whereas medians and quartiles were used for continuous variables (lack of normal distribution according to the Shapiro–Wilk test). The relationship between two categorical variables was tested using the chi-square test or Fisher’s exact test. The Mann–Whitney U test was used to compare differences between two independent groups for continuous variables. Correlations between numerical parameter values were determined on the basis of Spearman’s (R) rank correlation coefficient. Ventilator-associated pneumonia rate per 1000 ventilator days was estimated by dividing the number of VAP cases by the number of ventilator days and multiplying by 1000. Multivariable logistic regression was used to find the best fitting model to describe the relationship between: (i) VAP (the dependent variable) and both demographic and clinical factors; (ii) mortality and both demographic and clinical factors. Only independent variables with a *p*-value < 0.1 selected based on simple linear regression models were included in the backward step-wise regression. Regression coefficients (B), odds ratio (OR) and their 95% confidence intervals (CI) were demonstrated. All statistical tests were 2-tailed and a *p*-value equal to 0.05 or less was considered statistically significant in this study. STATISTICA v.13 (TIBCO Software Inc., Kraków, Poland, 2017) was used for statistical analysis.

## 3. Results

### 3.1. Demographic and Clinical Information of Patients Admitted to the ICU

Data were collected from 415 patients hospitalized in the ICU, and 371 patients (89.4%) were eligible for further analysis. A description of the cohort population is shown in Table 2. The median age of the cohort was 66 years (minimum 18 years, maximum 98 years), and 66.8% were male. The median APACHE II score on admission for the ICU was 23/71, and estimated risk of death was 46%. The patients were most frequently hospitalized for neurosurgical reasons (48.5%). Almost every fourth patient was diabetic (24.0%), and every fifth was diagnosed with chronic heart failure classified as NYHA ≥ 3 (19.7%) and was a nicotine smoker (22.1%). In total, 53.4% of patients received continuous control of endotracheal or tracheostomy tube cuff pressure together with continuous subglottic secretion suction. Multidrug-resistant (MDR) bacteria were found in the culture of lower respiratory secretions of 11.3% of patients. Almost every third patient had a tracheotomy performed, whereas enteral nutrition was provided to 90% of all patients (body mass index on admission for the ICU was 16–47 kg/m^2^). The median length of ventilator use was 8 [3; 17] days. Over 62% of patients were treated in the ICU longer than 5 days, and 44.7% of the patients died during their stay in the ward. The median age of the deceased group was significantly higher than the median age of the survivors (70 [61; 79] vs. 61 [49; 71]; Z = 5.43; *p* < 0.0001).

### 3.2. VAP Incidence and VAP Patient Characteristics

Among all 371 ventilation patients in the ICU, 52 (14%) developed VAP during their stay. The incidence density of VAP per 1000 ventilator days was 9.7. The median days from intubation to onset of VAP in the total cohort was 7 [4; 13], and in the retrospective cohort, this was significantly shorter than in the prospective cohort (5 [4; 11] vs. 9 [7; 18] days; Z = −2.09; *p* = 0.03). A majority of the patients had late-onset VAP (n = 35; 67.3%).

A subgroup analysis comparing patients with VAP and those without VAP showed that those with VAP had a statistically significant lower body mass index (24 vs. 26; Z = −2.51; *p* = 0.01) and were more likely to be admitted for neurosurgical reasons (61.5% vs. 46.4%; *χ*^2^ = 4.1; *p* = 0.04), had undergone a tracheostomy (69.2% vs. 24.1%; *χ*^2^ = 42.92; *p* < 0.001), had diagnosed urinary tract infection (28.8% vs. 12.8%; *χ*^2^ = 8.92; *p* = 0.003), had received enteral nutrition (96.1% vs. 85.3%; *χ*^2^ = 4.62; *p* = 0.03) and had been hospitalized longer than 5 days in the ICU (96.1% vs. 56.5%; *χ*^2^ = 29.56; *p* < 0.001).

### 3.3. Pathogens in VAP Patients

Multidrug-resistant bacteria were isolated more often in the culture of lower respiratory secretions of VAP patients than in patients without this infection (40.4% vs. 6.6%; *χ*^2^ = 50.88; *p* < 0.001). Prevalence of MDR pathogens were higher in late-onset VAP than in early-onset VAP (n = 18; 51.4% vs. n = 3; 17.6%; *χ*^2^ = 4.11; *p* = 0.03). Gram-negative bacteria species were the most common isolate. The study showed a higher frequency of Acinetobacter baumanii (15.4% vs. 3.3%), Pseumonas aeruginosa (15.4% vs. 0.3%), Klebsiella pneumoniae (Enterobacteriaceae; 28.8% vs. 8.1%) and other Enterobacteriaceae species (28.8% vs. 11.6%) in VAP patients compared with patients without VAP (*p* < 0.05). There was no significant difference in the distribution of the bacterial isolates between early- and late-onset VAPs (*p* > 0.05). The results of lower respiratory tract specimen cultures are presented in Table 3.

### 3.4. Factors Associated with VAP

According to the final model of multivariable logistic regression analysis, tracheotomy, MDR bacteria isolated in the culture of lower respiratory secretions and ICU length of stay >5 days were independent risk factors of VAP. On the other hand, the continuous control of cuff pressure and subglottic secretion suction used together were protective factors against VAP. Single-factor and multi-factor logistic regression models of VAP are showed in Table 4.

### 3.5. Length of Ventilator Use and ICU Length of Stay

VAP was found to be associated with an increased length of ventilator use and ICU length of stay. In VAP patients, the median time of mechanical ventilation (24 [11; 48] vs. 6 [3; 14] days; *Z* = 7.28; *p* < 0.0001) and stay in the ward (42 [18; 68] vs. 8 [3; 18] days; Z = 7.94; *p* < 0.0001) was significantly longer in comparison to non-VAP patients. A high positive correlation was found between length of ventilator use and ICU length of stay (*R* = 0.9; *t* = 47.39, *p* < 0.01). The presence of MDR pathogens in tracheal aspirates had no influence on the above-mentioned variables (*p* > 0.05).

### 3.6. Mortality

A smaller incidence of mortality (n = 16; 30.8% vs. n = 150; 47%; *χ*^2^ = 4.78; *p* = 0.03) was found in VAP patients compared to those without VAP. However, the estimated risk of death on admission to the ICU in the VAP group was 50.3% (APACHE II = 25 points), while in patients without VAP was 63.9% (APACHE II = 28 points). Factors significantly associated with mortality (Table 5) are age and APACHE II scores (both coefficients are positive) and enteral nutrition (negative regression coefficient).

## 4. Discussion

The study results indicated that a tracheotomy, MDR bacteria isolated in the culture of lower respiratory secretions and ICU length of stay more than 5 days were risk factors of VAP, whereas the implementation of continuous control of cuff pressure together with subglottic secretion suction were protective factors against VAP for patients who received mechanical ventilation.

These study findings showed that every seventh mechanically ventilated patient developed VAP. The incidence density of VAP per 1000 ventilator days in our study was lower than in the Polish ICU study carried out in 2007–2016 (9.7 vs. 15.2/1000 ventilation days) [24] and was similar to results of a study in the Netherlands (9.7 vs. 10.3/1000 ventilation days) [16]. Interpreting the above data, it should be taken into account that the diagnostic criteria used, the study period and the geographical area could affect the prevalence of VAP [25].

In the present study, a percutaneous tracheotomy was one of the significant factors associated with the development of VAP. This result appears consistent with previous findings obtained from a systematic review and meta-analysis conducted by Ding et al. [25]. A similar conclusion follows from a retrospective analysis carried out by Altinsoy et al. [26], who recorded VAP more frequently in patients who had undergone either a surgical or percutaneous tracheostomy. Some authors have suggested that for critically ill patients requiring prolonged mechanical ventilation, performing a tracheostomy within 10 days of admission can be associated with a shortened duration of mechanical ventilation [27]. In our study, patients had a tracheostomy performed between the 10th and 14th day after intubation. Unfortunately, the best timing for a tracheostomy still remains unclear, and any evidence of clinical benefits for an early tracheostomy (e.g., reduced incidence of mortality, duration of mechanical ventilation and length of stay in the ICU) is conflicting [28,29].

Previous studies have suggested that both patients with hospital-acquired pneumonia and those with VAP had a large burden of MDR pathogens [30], which were mainly associated with late-onset VAP [31,32]. Our results confirmed these reports. However, this study did not identify MDR organism infection as one of the risk factors for ICU mortality, which is in contrast with Chang et al. [30] or Feng et al. [33]. It is worth noting that the ICU mortality rate of our patients was connected with their age, health condition at the time of ICU admission and enteral nutrition. Similar to the findings of other studies, the higher the APACHE II score, the higher the mortality rate [34,35], while enteral nutrition was associated with a lower risk of death [36]. Data on the relationship between the age and mortality in patients treated with mechanical ventilation are conflicting [34,37]. In our study and in a meta-analysis carried out by Li et al. [38], VAP was not associated with an increased risk of mortality.

Both our studies and those conducted by others [31,39] found that Gram-negative bacteria were the predominant isolates from tracheal aspirates of VAP. In the present study, the most common etiologic agents responsible for VAP were Enterobacteriaceae species (including Klebsiella pneumonia) and non-fermenting Gram-negative bacteria (i.e., Acinetobacter baumannii and Pseudomonas aeruginosa). These results are in line with the previous findings of Hosamirudsari et al. [40] and Wałaszek et al. [9]. On the other hand, in the study conducted by Ben Lakha et al. [41], Acinetobacter baumanii was the most-common isolated organism, followed by Pseudomonas Aeruginosa and Enterobacterales. Similar to Xie et al. [8], we did not find any difference in the distribution of pathogens between early- and late-onset VAP.

Our observations confirmed that VAP is connected with prolonging duration of both mechanical ventilation and ICU stay [38,42]. On the other hand, we found that the chance of obtaining VAP increased with the extension of an ICU stay longer than 5 days. We hypothesized that the association between both variables may be a consequence of air contamination with a large number of pathogens. Monitoring of the levels of airborne microorganisms in an ICU by Dougall et al. [43] showed that air contamination levels increased with length of room occupancy and was correlated with ward activity. The afore-mentioned researchers indicated the following room activities which were linked to high levels of air contamination: presence of more than three staff, bed changes, patient personal hygiene, visiting hours, cleaning.

This study suggests that the continuous monitoring of cuff pressure of the artificial airway and continuous aspiration of subglottic secretion used together were protective factors against VAP. Akdogan et al. [44] reported that a decline in the VAP rate can be achieved thanks to the implementation of a VAP prevention bundle that includes oral hygiene with chlorhexidine, the manual control of cuff pressure (maintained at 20–30 mm Hg) and the aspiration of subglottic secretion. An overview of systematic reviews and an updated meta-analysis conducted by Pozuelo-Carrascosa et al. [1] also showed the positive effect of subglottic secretion drainage in reducing the incidence of VAP. Recent published studies have revealed that continuous control of cuff pressure significantly reduced the incidence of VAP when compared to intermittent methods [45], while continuous and intermittent subglottic secretion suction had a similar effect on the incidence of VAP [46]. However, these results are not conclusive, and large-scale multicenter, randomized clinical trials are needed to determine which continuous or intermittent technique is more beneficial as an intervention in the prevention of VAP.

## 5. Limitations and Strengths of the Study

Our work has several limitations. First, the generalizability of the study result is limited by the single-center study (the majority of patients were hospitalized for neurosurgical reasons). Second, data were collected both prospectively and retrospectively. Third, the use of continuous monitoring of cuff pressure of the artificial airway and aspiration of subglottic secretion from above the cuff along with other VAP preventive measures did not allow us to distinguish the individual effect of each of the above-mentioned methods to prevent VAP. Fourth, the cuff of standard endotracheal and tracheostomy tubes had a spherical or cylindrical shape and were made of polyvinyl chloride, while taper-shaped cuffs were made of polyurethane only in the case of tracheostomy tubes. However, a previously published study suggests that the shape of cuffs [47] and the type of material they consist of [48] have no influence on the incidence of VAP.

The major strength of the present study is the large-scale cohort. The study was developed according to STROBE guidelines, and the follow-up protocols were similar in the prospective and retrospective cohort.

## 6. Practical Implications of the Study

Increasingly better understanding of both VAP risk factors and interventions protecting against VAP should improve the quality of care for patients ventilated in the ICU. Our clinical practice indicates the need to prevent VAP by using both the pressure control in the cuff of endotracheal and tracheostomy tubes and the suction of the secretion from above the cuff of the tubes continuously. Maintaining constant pressure in the cuff, by reducing the number of cuff pressure drops <20 cm H_2_O [45], and thus limiting the micro-aspiration around the cuff, may reduce the incidence of VAP. In addition, the use of automated methods limits the nursing workload and/or increases the number of other nursing and therapeutic activities.

## 7. Conclusions

The results of this single-center cohort study indicated that for patients who received mechanical ventilation, a tracheotomy, MDR bacteria and ICU length >5 days were independent risk factors of VAP, whereas the implementation of continuous control of cuff pressure together with subglottic secretion suction were protective factors against VAP.

## Figures and Tables

**Figure 2:**
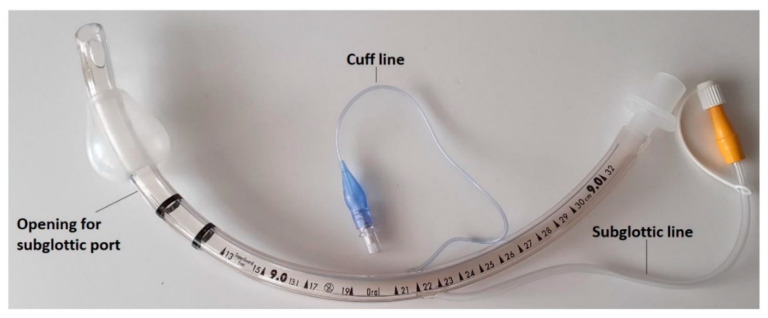
Endotracheal tube with a tapered cuff and channel for subglottic secretion suction.

**Figure 3:**
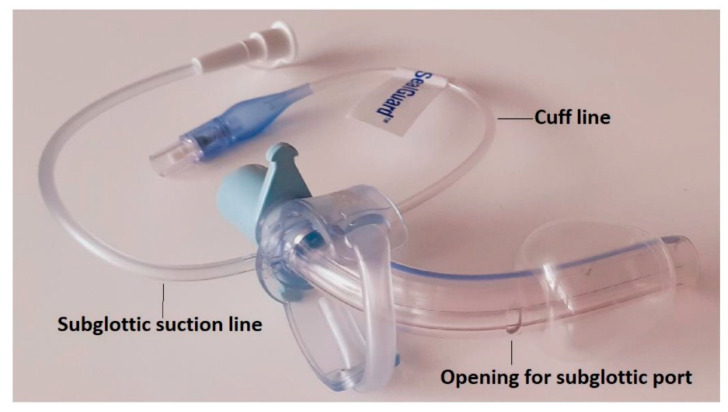
Tracheostomy tube with a tapered cuff and channel for subglottic secretion suction.

**Figure 4:**
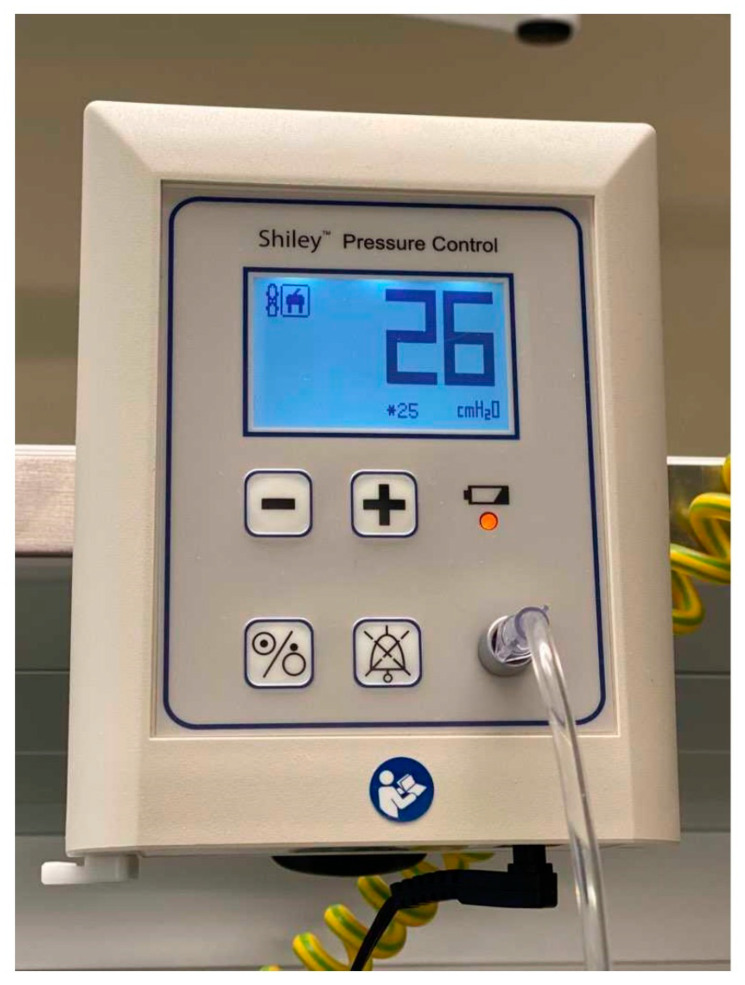
Shiley Pressure Control.

**Figure 5:**
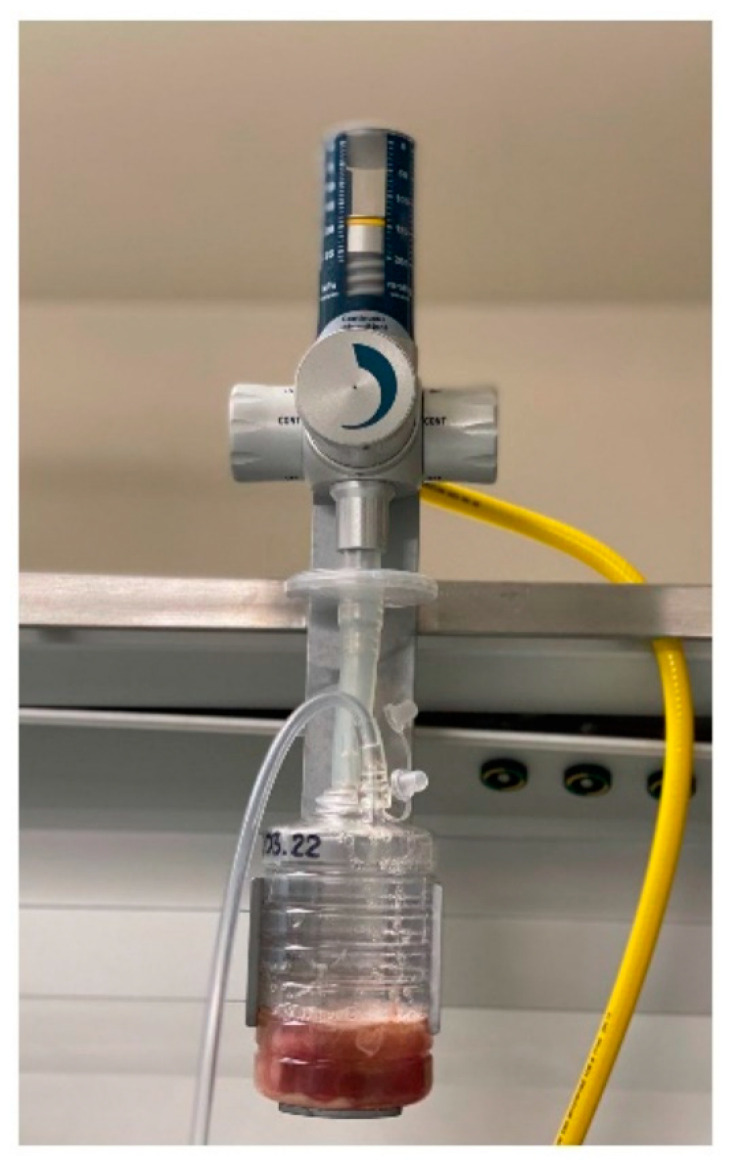
Hersill Vacusill 3 Continuous–Intermittent apparatus.

**Figure 6:**
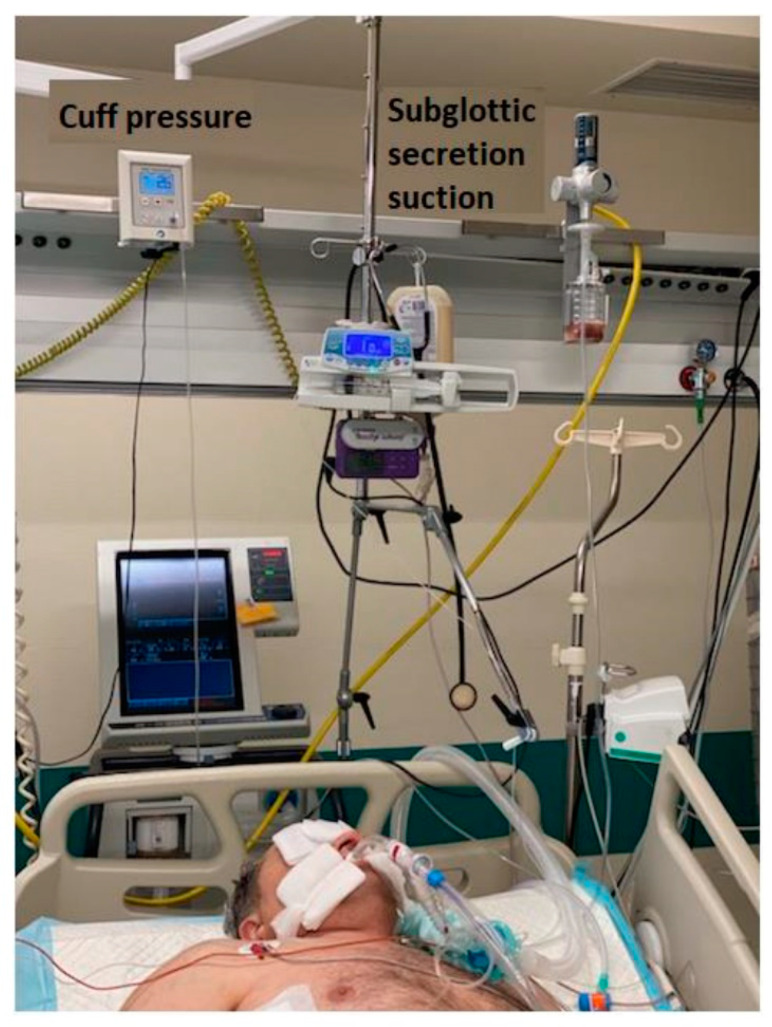
Pressure control and subglottic secretion suction used in patient with ventilator-associated pneumonia in the intensive care unit.

**Table 1:** VAP prevention bundle.

**The Prospective and Retrospective Cohort**	
Strict hand hygiene of medical staff. Head of bed elevation to 30–45 degrees—implemented already upon admission to the ICU, verified every 4 h. Daily sedation vacations (temporarily reducing sedation while the patient is ventilated) Moderately lung-sparing ventilation (VT 5–8 mL/kg due body weight, PEEP > 3 cm H_2_O with ventilator settings allow in for normocapnia and plateau pressure < 25 cm H_2_O) with rapid weaning from mechanical ventilation. Closed tracheal suction systems—replaced every 7 days or in the event of leakage. Oral hygiene with 0.2% chlorhexidine digluconate solution every 12 h. Peptic ulcer disease prophylaxis. Deep vein thrombosis prophylaxis.	
**The Prospective Cohort**	**The Retrospective Cohort**
9. Endotracheal tubes or tracheostomy tubes with a tapered cuff and channel for subglottic secretion suction (Figure 2 and Figure 3). 10. Cuff pressure control maintained at 25–30 mmHg—measured continuously and automatically ^1^ (Figure 4). 11. Aspiration of subglottic secretion—continuous ^2^, by suction tubing for evac aspiration ^3^, suction force: 100–150 mmHg (Figure 5 and Figure 6).	9. Standard endotracheal tubes or tracheostomy tubes without a channel for subglottic secretion suction. 10. Cuff pressure control maintained at 20–30 mmHg—measured with a manual manometer ^4^ (every 12 h or whenever hypotension/hypertension in the cuff was suspected).

^1^ Shiley Pressure Control, VBM Medizintechnik GmbH, Covidien, Germany; ^2^ Hersill Vacusill 3 Continuous–Intermittent apparatus (Madrid, Spain); ^3^ DAR™, Italy, ID 3.5 mm; ^4^ VBM Cuff Pressure Measuring, Germany.

**Table 2:** Baseline demographic and clinical characteristic of patients admitted to the ICU.

Demographic and Clinical Factors	*n* = 371
Age (years)	66 [54; 75]
Body mass index (kg/m^2^)	26 [23; 29]
APACHE II scores admission	23 [19; 28]
Estimated risk of death admission (%)	46 [32.2; 67.2]
Antibiotics prior to VAP	72 (19.4)
Sex	
Female	123 (33.1)
Male	248 (66.8)
Admission category	
Neurosurgical	180 (48.5)
Cardiovascular	77 (20.7)
General surgical	63 (17.0)
Non-cardiac internal medicine	51 (13.7)
Comorbiditie*s*	
Chronic heart failure	73 (19.7)
Chronic renal failure	35 (9.4)
Chronic liver failure	9 (2.4)
Chronic obstructive pulmonary disease	40 (10.8)
Gastric and duodenal ulcer	16 (4.3)
Diabetes mellitus	89 (24.0)
Immunosuppression	17 (4.5)
Smoking status	82 (22.1)
ICU stay	
Continuous control of cuff pressure and subglottic secretion suction used together	198 (53.4)
MDR pathogen in the culture of the lower respiratory secretions	42 (11.3)
Septic shock	70 (18.9)
Tracheotomy	113 (30.4)
Enteral nutrition	322 (86.8)
Length of ventilator use (day)	8 [3; 17]
ICU length of stay (day)	10 [4; 22]
ICU mortality	166 (44.7)

Categorical data were reported as total number and percentage; descriptive statistics are expressed as a median and upper and lower quartile; ICU = Intensive Care Units; APACHE II score = Acute Physiology and Chronic Health Evaluation II score; VAP = ventilator-associated pneumonia; MDR = multidrug-resistant bacteria.

**Table 3:** Pathogens isolated from patients with VAP and without VAP.

Pathogens	VAP (*n* = 52)	Non-VAP (*n* = 319)	*p* Value
Multidrug-resistant bacteria	21 (40.4)	21 (6.6)	<0.001
Gram-positive bacteria			
Methicyllin-resistant Staphylococcus aureus	2 (3.8)	4 (1.2)	0.17
Methicillin-sensitive Staphylococcus aureus	4 (7.7)	18 (5.6)	0.53
Staphylococcus epidermidis	4 (7.7)	18 (5.6)	0.53
Vancomycin-resistant enterococcus	1 (1.9)	1 (0.3)	0.26
Vancomycin-sensitive enterococcus	1 (1.9)	-	-
Gram-negative bacteria			
Acinetobacter baumanii	8 (15.4)	10 (3.3)	<0.001
Pseumonas aeruginosa	8 (15.4)	1 (0.3)	<0.001
Klebsiella pneumoniae (Enterobacteriaceae)	15 (28.8)	26 (8.1)	<0.001
Other Enterobacteriaceae species	15 (28.8)	37 (11.6)	<0.001
Haemophilus influenzae	2 (3.8)	14 (4.4)	0.61
Stenotrophomonas maltofila	3 (5.8)	15 (4.7)	0.73
Candida albicans	1 (1.9)	3 (0.9)	0.45

Data were reported as total number and percentage; VAP = ventilator-associated pneumonia.

**Table 4:** Single-factor and multi-factor logistic regression model of VAP patients who received mechanical ventilation.

Factors	B	SE	Wald	OR	95% CI	*p* Value
	Simple logistic regression					
Admission category—neurosurgical	0.31	0.15	4.02	1.36	1.01–1.84	0.04
Urinary tract infection	0.51	0.17	8.40	1.66	1.18–2.33	0.004
Enteral nutrition	0.73	0.37	3.93	2.08	1.01–4.28	0.048
Tracheotomy	0.98	0.16	35.63	2.66	1.93–3.67	<0.001
Multidrug-resistant bacteria ^1^	1.13	0.18	39.14	3.10	2.17–4.42	<0.001
ICU length of stay >5 days	1.47	0.36	16.31	4.37	2.13–8.93	<0.001
Body mass index	−0.08	0.03	6.27	0.92	0.86–0.98	0.01
Continuous control of cuff pressure and subglottic secretion suction used together	−0.40	0.15	6.65	0.67	0.50–0.91	0.01
	Multivariable logistic regression model ^2^					
Tracheotomy	0.47	0.19	6.23	1.60	1.10–2.31	0.01
Multidrug-resistant bacteria ^1^	1.0	0.20	24.14	2.73	1.83–4.07	<0.001
ICU length of stay >5 days	1.2	0.39	9.27	3.32	1.53–7.19	0.002
Continuous control of cuff pressure and subglottic secretion suction used together	−0.50	0.18	7.55	0.61	0.43–0.87	0.006

B = regression coefficient; SE = Standard error; OR = Odds ratio; CI = Confidence interval; ^1^ Isolated in the culture of the lower respiratory secretions; ^2^ Standard measures of goodness: R2 Nagelkerka = 0.36; Hosmer Lemeshow = 2.90, *p* = 0.72.

**Table 5:** Single-factor and multi-factor logistic regression model of mortality.

Factors	B	SE	Wald	OR	95% CI	*p* Value
	Simple logistic regression					
Age	0.04	0.01	26.37	1.04	1.02–1.06	<0.0001
APACHE II admission	0.16	0.02	63.03	1.18	1.13–1.23	<0.001
Smoking status	0.29	0.13	5.41	1.34	1.05–1.72	0.02
Enteral nutrition	−0.59	0.16	12.84	0.55	0.40–0.76	0.0003
	Multivariable logistic regression model ^1^					
Age	0.02	0.01	4.91	1.02	1.00–1.04	0.03
APACHE II admission	0.14	0.02	43.6	1.16	1.12–1.23	<0.001
Enteral nutrition	−0.46	0.20	5.43	0.63	0.43–0.93	0.02

B = regression coefficient; SE = Standard error; OR = Odds ratio; CI = Confidence interval; ^1^ Standard measures of goodness: R2 Nagelkerka = 0.30, Hosmer Lemeshow = 11.77, *p* = 0.16.

## Data Availability

A dataset will be made available upon request to the corresponding authors 1 year after the publication of this study. The request must include a statistical analysis plan.
